# CT-defined sarcopenia and outcomes in HR+/HER2− metastatic breast cancer treated with CDK4/6 inhibitors

**DOI:** 10.3389/fmed.2026.1943515

**Published:** 2026-10-28

**Authors:** Zeynep Alaca Topcu, Zehra Sucuoglu Isleyen, Ismail Yurtsever, Bugra Oztosun, Engin Erdemoglu, Mehmet Besiroglu, Haci Mehmet Turk, Mahmut Gumus

**Affiliations:** 1 Istanbul Medeniyet University Göztepe Prof Dr Süleyman Yalçın City Hospital, Department of Medical Oncology, İstanbul, Türkiye; 2 Bezmialem Vakif University Hospital, Department of Medical Oncology, İstanbul, Türkiye; 3 Bezmialem Vakif University Hospital, Department of Radiology, İstanbul, Türkiye

**Keywords:** CDK4/6 inhibitors, HR-positive/HER2-negative metastatic breast cancer, progression-free survival, sarcopenia, skeletal muscle index

## Abstract

**Background:**

Sarcopenia is increasingly recognized as a potential prognostic factor in patients with cancer, but its clinical significance in HR+/HER2− metastatic breast cancer treated with CDK4/6 inhibitors remains uncertain. This study investigated the association between CT-defined sarcopenia and clinical outcomes in this population.

**Methods:**

This retrospective multicenter study included 94 patients with HR+/HER2− metastatic breast cancer who received ribociclib or palbociclib plus endocrine therapy between January 2021 and March 2024. Baseline sarcopenia was defined using the skeletal muscle index measured on CT at the third lumbar vertebral level (L3). Progression-free survival (PFS) was estimated using the Kaplan–Meier method. Cox proportional hazards regression was used to identify prognostic factors for PFS, with CT-defined sarcopenia, continuous skeletal muscle index (SMI), and total muscle area (TMA) evaluated in separate multivariable models.

**Results:**

CT-defined sarcopenia was present in 37 patients (39.4%). Median PFS was markedly shorter in patients with sarcopenia than in those without sarcopenia (15.2 vs. 26.6 months; log-rank *p* < 0.001). At 1 and 2 years, PFS rates were 57.6% and 15.2% in patients with sarcopenia versus 84.1% and 56.0% in those without sarcopenia, respectively. In separate multivariable models, CT-defined sarcopenia remained independently associated with shorter PFS (HR, 3.22; 95% CI, 1.86–5.59; *p* < 0.001), whereas higher continuous SMI (HR, 0.93; 95% CI, 0.89–0.97; *p* = 0.001) and TMA (HR, 0.97; 95% CI, 0.96–0.99; *p* = 0.002) were independently associated with longer PFS. Higher Ki-67 expression and second-line CDK4/6 inhibitor therapy also remained significantly associated with shorter PFS across all three models.

**Conclusions:**

CT-defined sarcopenia was an independent prognostic factor of inferior PFS in patients with HR+/HER2− metastatic breast cancer receiving CDK4/6 inhibitor-based therapy. Assessment of skeletal muscle mass on routinely acquired baseline CT scans may offer a practical tool for risk stratification and may help identify patients requiring more intensive follow-up and supportive care.

## 1 Introduction

Hormone receptor-positive/human epidermal growth factor receptor 2-negative (HR+/HER2−) breast cancer is the most common subtype of breast cancer, accounting for approximately 70% of all cases (1). Endocrine therapy combined with CDK4/6 inhibition has become the standard treatment for HR+/HER2− metastatic breast cancer, based on the substantial PFS and OS benefits demonstrated over the past decade (2–5). Despite these advances, clinical outcomes remain heterogeneous, highlighting the need for reliable biomarkers for risk stratification and prognostic assessment.

Body composition parameters have gained increasing attention as potential prognostic markers in oncology. Among these, sarcopenia, characterized by the progressive loss of skeletal muscle mass and function, has been associated with impaired physical performance, greater treatment-related toxicity, poorer treatment tolerance, diminished quality of life, and unfavorable survival outcomes across multiple malignancies (6–10). In breast cancer, sarcopenia has similarly been associated with poorer survival and clinical outcomes (11–15). Skeletal muscle depletion may reflect the complex interplay among systemic inflammation, metabolic dysregulation, malnutrition, and cancer-related cachexia, potentially contributing to tumor progression and resistance to anticancer therapy (16, 17). Computed tomography (CT)-based assessment of skeletal muscle mass provides an accurate and clinically accessible approach to identifying sarcopenia as CT scans are routinely obtained for staging and response assessment in metastatic cancer (6, 18).

The management of HR+/HER2− metastatic breast cancer has substantially improved with the introduction of CDK4/6 inhibitors; however, the prognostic significance of sarcopenia in this patient population has not yet been clearly established. Studies examining the association between sarcopenia and outcomes with CDK4/6 inhibitor therapy have reported inconsistent findings, particularly regarding survival and treatment-related toxicity (19–21). Nevertheless, several studies have demonstrated significantly shorter PFS and OS in patients with sarcopenia than in those without sarcopenia (22–25). Given the prolonged duration of CDK4/6 inhibitor therapy and the importance of preserving nutritional and functional status, clinically accessible prognostic biomarkers may improve risk stratification and support individualized patient management. Accordingly, this study evaluated the association between CT-defined sarcopenia and clinical outcomes in patients with HR+/HER2− MBC treated with CDK4/6 inhibitors.

## 2 Methods

### 2.1 Study population and data collection

This retrospective cohort study was conducted at two tertiary oncology centers. Patients aged ≥18 years with hormone receptor-positive (HR+)/HER2-negative metastatic breast cancer, including *de novo* metastatic or recurrent disease, were eligible if they received first- or second-line ribociclib or palbociclib plus an aromatase inhibitor or fulvestrant between January 2021 and March 2024. Eligibility also required baseline abdominal CT or PET/CT imaging performed within 30 days before treatment initiation for sarcopenia assessment. Exclusion criteria were HER2-positive disease, unavailable or inadequate baseline imaging for sarcopenia assessment, Eastern Cooperative Oncology Group performance status (ECOG PS) >2, and concurrent malignancy. The patient flow diagram is shown in Figure 1.

**Figure 1:**
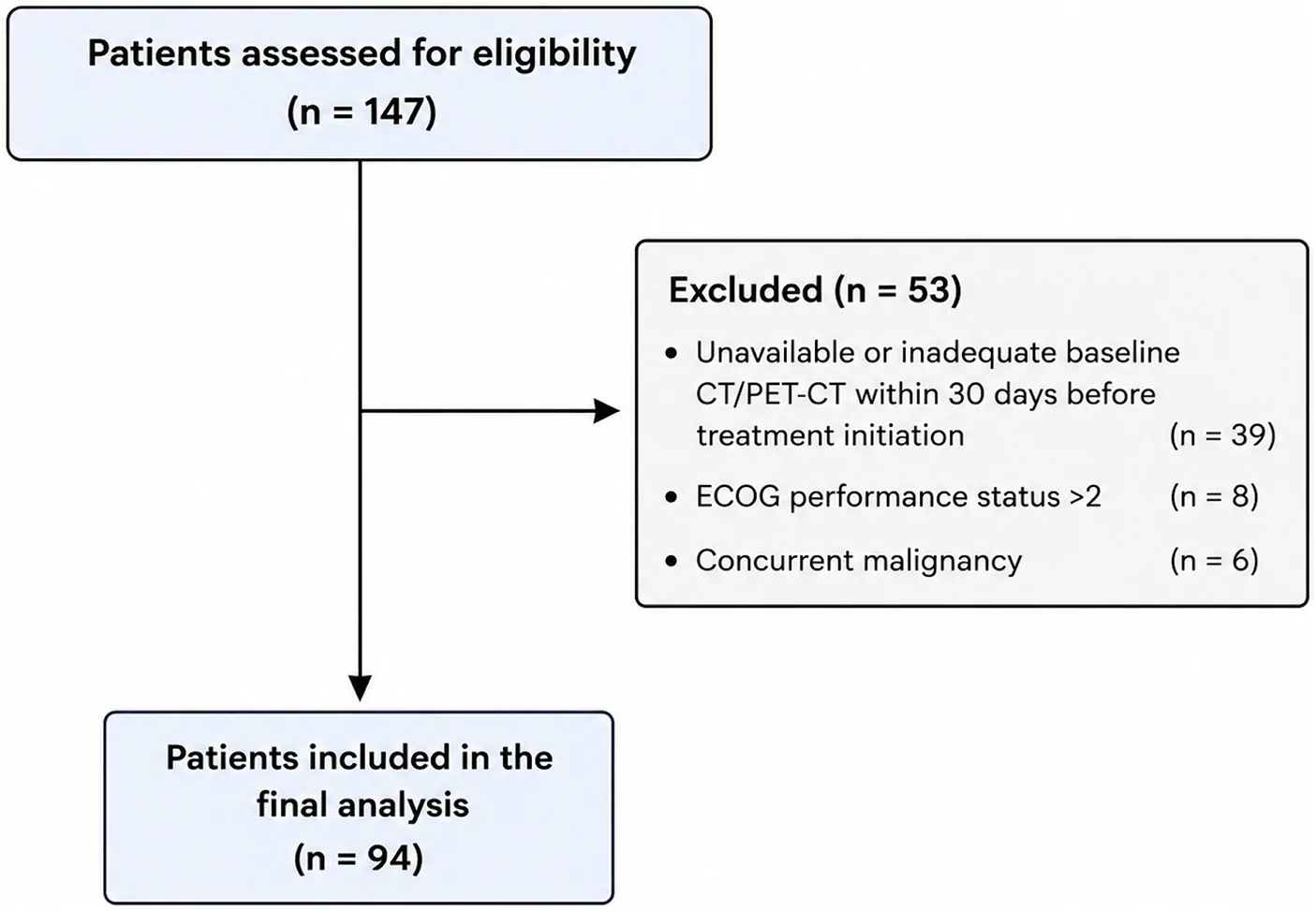
The patient flow diagram.

Clinical, demographic, pathological, treatment-related, and outcomes were assessed retrospectively. Hormone receptor and HER2 status were determined from pathological reports. HR-positive disease was defined as estrogen receptor and/or progesterone receptor expression in ≥1% of tumor cells. HER2-negative disease was defined as an immunohistochemistry score of 0 or 1+, or a score of 2 + with negative fluorescence *in situ* hybridization (FISH).

### 2.2 Treatment

All patients received ribociclib or palbociclib plus endocrine therapy with letrozole or fulvestrant. Abemaciclib was not used because it was not reimbursed in Türkiye during the study period. Ribociclib and palbociclib were initiated at standard doses of 600 mg and 125 mg, respectively, for 21 consecutive days followed by a 7-day break, with continuous endocrine therapy. Dose reductions and temporary treatment interruptions were implemented when clinically indicated according to standard toxicity-management recommendations.

Clinical assessments were performed approximately every 12 weeks. Radiological assessments were conducted primarily using contrast-enhanced thoracoabdominopelvic CT, with PET/CT performed when clinically indicated. Tumor response was evaluated according to the Response Evaluation Criteria in Solid Tumors (RECIST) version 1.1 (26) and classified as complete response (CR), partial response (PR), stable disease (SD), or progressive disease (PD). Objective response rate (ORR) comprised patients with CR or PR, whereas disease control rate (DCR) comprised those with CR, PR, or SD. Response assessments were performed by treating physicians during routine clinical follow-up. Progression-free survival (PFS) was defined as the time from CDK4/6 inhibitor initiation to documented disease progression or death from any cause, whichever occurred first.

### 2.3 CT-based assessment of sarcopenia

Baseline abdominal CT images obtained within 30 days before CDK4/6 inhibitor initiation as part of routine staging were used for skeletal muscle assessment. No additional imaging was performed for this study. All scans were reviewed by a single experienced radiologist who was blinded to the patients’ clinical, laboratory, and pathological data.

Skeletal muscle area was measured on a single contrast-enhanced axial CT image at the level of the third lumbar vertebra (L3), selected with both transverse processes clearly visible, using the Syngo.via workstation (software version VB30A_HF06; Siemens Healthineers). For patients evaluated with PET/CT, the CT component of the examination was used for analysis. Skeletal muscle was identified using an attenuation range of −29 to +150 Hounsfield units (HU). The psoas, paraspinal, and abdominal wall muscles were included, and the measured area was recorded as total muscle area (TMA, cm^2^). The skeletal muscle index (SMI, cm^2^/m^2^) was calculated by normalizing TMA to height squared (SMI = TMA/height^2^) (6). CT-defined sarcopenia was defined using sex-specific L3 SMI thresholds of ≤39 cm^2^/m^2^ for women and ≤55 cm^2^/m^2^ for men, based on established criteria (13, 16, 27).

Because muscle strength and physical performance data were unavailable in this retrospective study, sarcopenia was not definitively established based on EWGSOP2 criteria; therefore, the term “CT-defined sarcopenia” was used throughout the study (6).

### 2.4 Statistical analysis

Statistical analyses were performed using IBM SPSS Statistics version 25.0 (IBM Corp., Armonk, NY, USA). Categorical variables are presented as frequencies and percentages, and continuous variables as mean ± standard deviation or median (range), as appropriate. Normality was assessed using the Kolmogorov–Smirnov test. Categorical variables were compared using the Pearson chi-square or Fisher's exact test, as appropriate. Continuous variables were compared using the independent-samples t-test or Mann–Whitney U test based on data distribution.

PFS survival was estimated using Kaplan–Meier method and compared across groups using the log-rank test. Prognostic factors for PFS were assessed using univariable and multivariable Cox proportional hazards regression analyses. Variables with *p* < 0.05 in univariable analysis, together with age and ECOG PS based on their clinical relevance, were considered for in the multivariable analysis. To avoid collinearity, CT-defined sarcopenia, SMI, and TMA were evaluated in three separate multivariable. Cox regression models. Given the potential overlap between treatment line and endocrine partner, treatment line was retained as the broader measure of treatment history to maintain model parsimony. Each model included the respective skeletal muscle variable (CT-defined sarcopenia, SMI, or TMA), age, ECOG PS, progesterone receptor level (PgR), Ki-67 level, and treatment at second line. Age and ECOG PS were retained in all models irrespective of statistical significance because of their clinical relevance. Hazard ratios (HRs) and 95% confidence intervals (CIs) were reported. The proportional hazards assumption was assessed using Schoenfeld residuals. All tests were two-sided, and *p* < 0.05 was considered statistically significant.

### 2.5 Ethical considerations

Access to patient data was restricted to the investigators involved in data collection, analysis, and manuscript preparation, in accordance with institutional confidentiality policies. The study was conducted in accordance with institutional and international ethical standards, including the Declaration of Helsinki. Ethical approval was obtained from the Clinical Research Ethics Committee of Bezmialem Vakif University (approval no. 2022/396).

## 3 Results

Of the 94 patients, 37 (39.4%) had CT-defined sarcopenia and 57 (60.6%) did not. The mean age was 54.9 years, and 91 patients (96.8%) were women. Baseline clinical, pathological, and body composition characteristics are summarized in Table 1.

**Table 1:** Baseline characteristics according to CT-defined sarcopenia status (*n* = 94).

Characteristics	Total (n, %)	Sarcopenic (n, %)	Non-Sarcopenic (n, %)	*p*-value
Age (mean ± SD)	54.94 ± 13.29	55.95 ± 14.58	54.28 ± 12.47	0.556
BMI (kg/m^2^)	28.64 ± 5.7	27.14 ± 5.69	29.62 ± 5.55	**0.040**
**Gender**				
Female	91 (96.8%)	35 (38.5%)	56 (61.5%)	0.325
Male	3 (3.2%)	2 (2.1%)	1 (1.8%)	
**Age**				
<65	68 (72.3%)	27 (28.7%)	41 (43.6%)	0.912
≥65	26 (27.7%)	10 (10.6%)	16 (17.0%)	
**ECOG PS**				
0–1	82 (87.2%)	31 (33.0%)	51 (54.0%)	0.419
2	12 (12.8%)	6 (6.4%)	6 (6.4%)	
**BMI**				
≥30	35 (37.2%)	8 (8.5%)	27 (28.7%)	**0.012**
<30	59 (62.8%)	29 (33.9%)	30 (31.9%)	
**Metastasis**				
*De novo*	45 (47.9%)	18 (19.1%)	27 (28.7%)	0.903
Metachronous	49 (52.1%)	19 (20.2%)	30 (31.9%)	
**Smoking History**				
Yes	15 (16%)	7 (7.4%)	8 (8.5%)	0.528
No	79 (84%)	30 (31.9%)	49 (52.1)	
**At least ≥1 comorbidity**				
Yes	34 (36.2%)	15 (16.0%)	19 (23.4%)	0.477
No	60 (63.8%)	22 (23.4%)	38 (43.4%)	
**Grade**				
1–2	48 (53.4%)	23 (25.6%)	25 (27.8%)	0.101
3	42 (46.6%)	13 (14.4%)	29 (32.2%)	
**Histology**				
Ductal	81 (86.2%)	31 (33%)	50 (53.2%)	0.589
Lobuler	13 (13.8%)	6 (6.4%)	7 (7.4%)	
**Menopausal Status**				
Pre/perimenopausal	36 (38.3%)	13 (13.8%)	23 (24.5%)	0.611
Postmenopausal	58 (61.7%)	24 (25.5%)	34 (36.2%)	
**Line of CDK4/6 inhibitor**				
First Line	54 (57.4%)	20 (21.3%)	34 (36.2%)	0.592
Second Line	40 (42.6%)	17 (18.1%)	23 (24.5%)	
**CDK4/6 inhibitor**				
Ribociclib	76 (80.9%)	30 (31.9%)	46 (48.9%)	0.964
Palbociclib	18 (19.1%)	7 (7.4%)	11 (11.7%)	
**Endocrine treatment**				
Aromatase inhibitor	53 (56.4%)	18 (19.1%)	35 (37.2%)	0.223
Fulvestrant	41 (43.6%)	19 (20.2%)	22 (23.4%)	
**Surgery before CDK4/6 inhibitor**				
Yes	54 (57.4%)	21 (22.3%)	33 (35.2%)	0.913
No	40 (42.6%)	16 (17%)	24 (25.5%)	
**Brain Metastasis**				
Yes	8 (8.5%)	3 (3.2%)	5 (5.3%)	0.913
No	86 (91.5%)	34 (36.2%)	52 (55.3%)	
**Liver Metastasis**				
Yes	20 (21.3%)	10 (10.6%)	10 (10.6%)	0.272
No	74 (78.7%)	27 (28.7%)	47 (50%)	
**Lung Metastasis**				
Yes	37 (39.4%)	15 (16.0%)	22 (23.4%)	0.850
No	57 (60.6%)	22 (23.4%)	35 (37.2%)	
**Bone Metastasis**				
Yes	82 (87.2%)	35 (38.5%)	47 (50%)	0.085
No	12 (12.8%)	2 (2.1%)	10 (10.6%)	
**Metastatic Organs**				
1	37 (39.4%)	13 (13.8%)	24 (25.5%)	0.913
>1	57 (60.6%)	24 (25.5%)	33 (35.2%)	
**TMA (cm^2^)**	102.85 (IQR 91.25–117.15)	89.0 (IQR 79.70–94.70)	110.60 (IQR 104.20–121.62)	**0.001**
**SMI (cm^2^/m^2^)**	41.00 (IQR 36.91–46.15)	36.11 (IQR 31.51–38.12)	43.57 (IQR 41.45–49.03)	**0.001**
**Estrogen Receptor (ER) (%)**	95 (IQR 90–95)	90 (IQR 80–95)	95 (IQR 90–95)	**0.010**
**Progesterone Receptor (PgR) (%)**	55 (IQR 10–80)	20 (IQR 5–80)	60 (IQR 20–80)	**0.040**
**Ki-67 (%)**	30 (IQR 15–40)	30 (IQR 12–40)	30 (IQR 18–40)	**0.807**

Histological grade data were unavailable for 4 patients (*n* = 90); all other variables were available for the entire cohort (*n* = 94).

BMI (kg/m^2^), body mass index; ECOG PS, Eastern Cooperative Oncology Group Performance Status; TMA, total muscle area; CDK, Cyclin-dependent kinase; SMI, skeletal muscle index.

After a median follow-up of 35.8 months (95% CI, 30.7–41.0), median PFS was 20.0 months (95% CI, 15.5–24.6). Patients with CT-defined sarcopenia had significantly shorter median PFS than those without it: 15.2 versus 26.6 months (95% CI, 11.0–19.4 and 21.2–32.1, log-rank *p* < 0.001). The PFS rates at 1 and 2 years were 57.6% and 15.2%, respectively, in patients with sarcopenia, compared with 84.1% and 56.0% in those without sarcopenia. Kaplan–Meier curves for patients with and without sarcopenia are shown in Figure 2.

**Figure 2:**
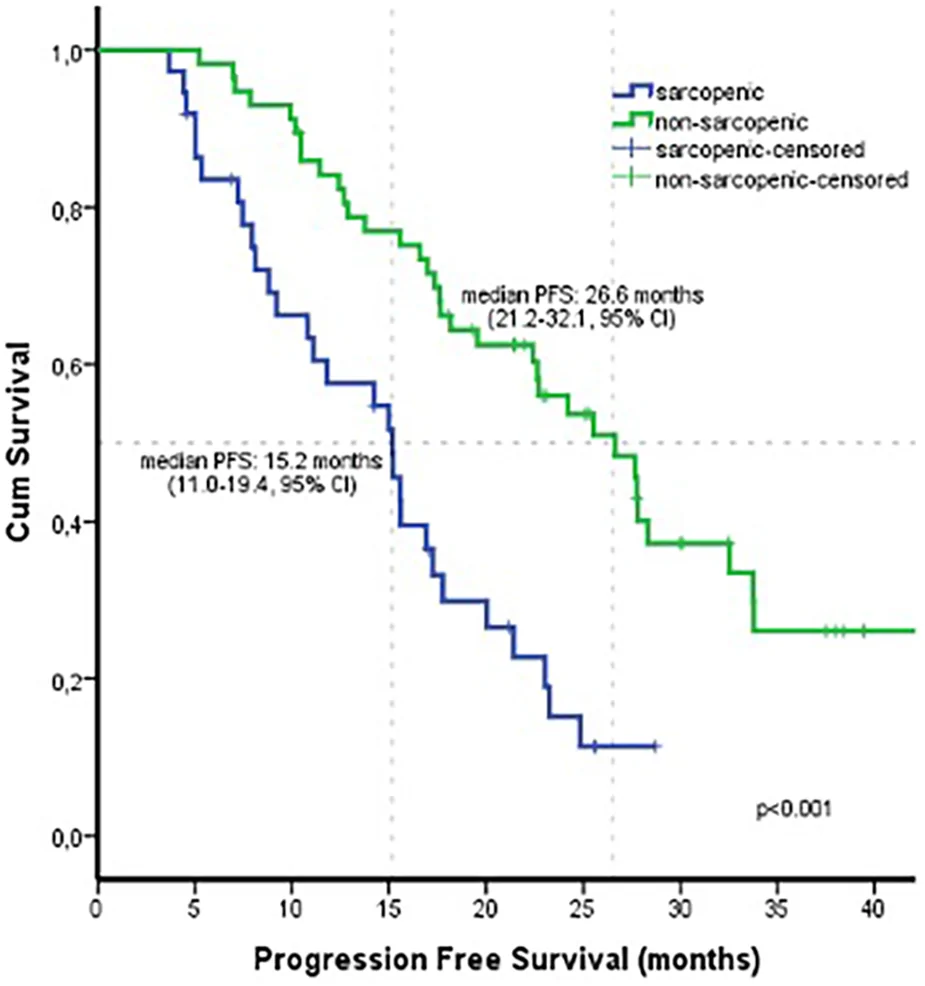
Progression-free survival by CT-defined sarcopenia status

At the first radiological assessment, performed approximately 3 months after treatment initiation, CR was observed in 6 patients (6.4%), PR in 73 (77.7%), SD in 10 (10.6%), and PD in 5 (5.3%), resulting in an ORR of 84.0% and a DCR of 94.7%. Best overall response was CR in 22 patients (23.4%), PR in 58 (61.7%), SD in 9 (9.6%), and PD in 5 (5.3%), with an ORR of 85.1% and a DCR of 94.7%. Disease progression was observed in 63 patients (67.0%), whereas 31 (33.0%) remained progression-free at the last assessment.

In univariable Cox regression analysis, greater TMA (HR, 0.97; 95% CI, 0.96–0.99; *p* = 0.001), greater SMI (HR, 0.94; 95% CI, 0.90–0.97; *p* = 0.002), and higher progesterone receptor expression (HR, 0.99; 95% CI, 0.98–0.99; *p* = 0.033) were associated with longer PFS. In contrast, CT-defined sarcopenia (HR, 2.87; 95% CI, 1.70–4.86; *p* < 0.001), second-line CDK4/6 inhibitor therapy (HR, 2.01; 95% CI, 1.28–3.46; *p* = 0.003), higher Ki-67 expression (HR, 1.01; 95% CI, 1.00–1.03; *p* = 0.005), and fulvestrant-based endocrine therapy (HR, 2.17; 95% CI, 1.32–3.58; *p* = 0.002) were linked to shorter PFS (Table 2).

**Table 2:** Results of univariate analyses of CDK4/6 progression-free survival (PFS).

	Univariate analysis	
Variables	HR (95% Cl)	*p*-value
Male gender	0.92 (0.22–3.78)	0.910
Age >65 years	0.66 (0.36–1.20)	0.171
Smoking History	0.68 (0.32–1.43)	0.314
≥1 comorbidity	0.94 (0.55–1.59)	0.824
BMI ≥30 kg/m^2^	1.43 (0.87–2.36)	0.154
ECOG PS 2	0.36 (0.11–1.16)	0.088
TMA (cm^2^)	**0.97** (**0.96–0.99)**	**0.001**
SMI (cm^2^/m^2^)	**0.94** (**0.90–0.97)**	**0.002**
CT-defined sarcopenia	**2.87** (**1.70–4.86)**	**<0.001**
Postmenopausal status	1.11 (0.67–1.85)	0.671
Grade 3	1.57 (0.94–2.61)	0.079
Lobuler histology	0.73 (0.34–1.54)	0.410
ER level	0.98 (0.96–1.00)	0.075
PgR level	**0.99** (**0.98–0.99)**	**0.033**
Ki-67 level	**1.01** (**1.00–1.03)**	**0.005**
Metachronous metastases	1.44 (0.87–2.39)	0.152
Treatment at second line	**2.01** (**1.28–3.46)**	**0.003**
Palbociclib	0.59 (0.28–1.26)	0.177
Fulvestrant	**2.17** (**1.32–3.58)**	**0.002**
Brain metastasis	1.74 (0.79–3.84)	0.195
Liver metastasis	1.59 (0.91–2.78)	0.102
Lung metastasis	0.60 (0.35–1.03)	0.067
Bone metastasis	1.96 (0.88–4.32)	0.096
Metastatic organs (more than one site)	1.32 (0.79–2.20)	0.282
Breast surgery history	1.26 (0.75–2.09)	0.374
Hemoglobin (<10 gr/dL)	0.93 (0.40–2.18)	0.883
Albumin (<3.5 gr/dL)	1.13 (0.15–8.21)	0.904
CRP (>10 mg/L)	0.64 (0.33–1.25)	0.194

HR, hazard ratio; ECOG PS, Eastern Cooperative Oncology Group Performance Status; CDK, cyclin-dependent kinase; CT, computed tomography; BMI, Body mass index; TMA, total muscle area; CRP, C-Rea**c**tive Protein; ER, estrogen receptor; PgR, progesterone receptor; SMI, skeletal muscle index.

To avoid collinearity among CT-defined sarcopenia, SMI, and TMA, three separate multivariable Cox regression models were constructed (Table 3). In Model 1, CT-defined sarcopenia remained independently associated with shorter PFS (HR, 3.22; 95% CI, 1.86–5.59; *p* < 0.001). In Model 2, higher SMI was independently associated with longer PFS (HR, 0.933; 95% CI, 0.895–0.973; *p* = 0.001), while in Model 3, higher TMA was independently associated with longer PFS (HR, 0.976; 95% CI, 0.960–0.991; *p* = 0.002). Higher Ki-67 expression and second-line CDK4/6 inhibitor therapy remained significantly associated with shorter PFS across all three models. Age, ECOG performance status, and PgR level were not statistically significant in the multivariable models (Table 3). No significant violation of the proportional hazards assumption was observed.

**Table 3:** Multivariable cox regression models for progression-free survival (PFS).

Variable	Model-1: Sarcopenia HR (95% CI), *p* value	Model-2: SMI HR (95% CI), *p* value	Model-3: TMA HR (95% CI), *p* value
CT-defined sarcopenia	**3.22 (1.86–5.59), <0.001**	-	-
SMI (cm^2^/m^2^)	-	**0.93 (0.89–0.97), 0.001**	**-**
TMA (cm^2^)	-	-	**0.97 (0.96–0.99), 0.002**
Age	1.01 (0.98–1.03), 0.403	1.012 (0.989–1.035), 0.316	1.007 (0.987–1.029), 0.565
PgR level	0.999 (0.991–1.007), 0.783	0.997 (0.990–1.005), 0.488	0.997 (0.990–1.004), 0.426
Ki67 level	**1.019 (1.004–1.034), 0.011**	**1.019 (1.004–1.034), 0.014**	**1.021 (1.006–1.037), 0.006**
ECOG	0.23 (0.05–1.02), 0.054	0.278 (0.06–1.21), 0.090	0.33 (0.07–1.45), 0.145
Treatment at second line	**2.05 (1.23–3.44), 0.006**	**2.43 (1.44–4.10), 0.001**	**2.22 (1.32–3.73), 0.003**

HR, hazard ratio; ECOG PS, Eastern Cooperative Oncology Group Performance Status; CDK, Cyclin-Dependent Kinase; CT, computed tomography; TMA, total muscle area; PgR, progesterone receptor, SMI, skeletal muscle index.

## 4 Discussion

CT-defined sarcopenia was present in 39.4% of this real-world cohort and was associated with significantly shorter PFS. Median PFS was markedly shorter in sarcopenic than non-sarcopenic patients, and CT-defined sarcopenia remained independently associated with shorter PFS in multivariable analysis. Because skeletal muscle assessment can be performed using CT images routinely obtained for staging and treatment monitoring, CT-defined sarcopenia may provide clinically relevant and readily accessible prognostic information beyond conventional clinicopathological factors in this setting.

Our findings are consistent with evidence linking CT-derived skeletal muscle measures to clinical outcomes. In a large cohort with early-stage disease, Caan et al. reported that CT-defined sarcopenia was associated with increased mortality and provided prognostic information beyond BMI in breast cancer (19). Systematic reviews have similarly associated sarcopenia with poorer outcomes and greater treatment-related toxicity, although substantial heterogeneity remains across studies. Consistent with this heterogeneity, Jang et al. emphasized the uncertainty surrounding the prognostic value of sarcopenia in metastatic breast cancer and highlighted the need for treatment-specific studies stratified by molecular subtype and systemic therapy (14). The present study addresses this gap by focusing exclusively on patients receiving CDK4/6 inhibitor-based therapy, the established treatment standard for HR+/HER2− metastatic breast cancer. Studies in this population have reported associations between baseline sarcopenia or reduced skeletal muscle index and shorter PFS (22–25). Our findings are consistent with these reports and support CT-defined sarcopenia as an accessible and objective imaging marker for risk stratification in this population.

Several mechanisms may underlie the association between sarcopenia and poorer outcomes. Skeletal muscle depletion may reflect systemic inflammation, impaired nutritional reserve, reduced physical activity, and cancer-related metabolic dysregulation, potentially compromising treatment tolerance, altering the pharmacokinetics of orally administered targeted therapies, and accelerating functional decline. BMI alone may not adequately capture body composition abnormalities, as patients with normal or elevated BMI may still have reduced skeletal muscle mass. In our cohort, although BMI was lower in patients with sarcopenia, obesity was also present in a subset, supporting the added value of CT-based skeletal muscle assessment beyond BMI alone (7, 16, 18, 28).

Although EWGSOP2 identifies low muscle strength as the primary feature of sarcopenia, CT is a reference-standard method for assessing skeletal muscle quantity. Because muscle strength and physical performance data were unavailable in this retrospective study, the term “CT-defined sarcopenia” was used (6, 18). These findings reflect imaging-defined muscle depletion rather than clinically diagnosed sarcopenia.

Importantly, the association between skeletal muscle mass and PFS remained consistent across the three separate multivariable models. CT-defined sarcopenia was independently associated with shorter PFS, whereas higher continuous SMI and TMA were independently associated with longer PFS when evaluated in separate models. These findings demonstrate the consistency of the association across categorical and continuous measures of skeletal muscle mass while avoiding collinearity among closely related body-composition variables. The multivariable modeling strategy was revised to address collinearity among closely related body-composition variables and to improve model parsimony; importantly, the association between CT-defined sarcopenia and shorter PFS remained consistent across the original and revised model specifications.

In addition to sarcopenia, higher Ki-67 expression and second-line CDK4/6 inhibitor therapy were independently associated with shorter PFS. The association with Ki-67 is biologically plausible, as greater proliferative activity may reflect more aggressive tumor biology (29, 30). The poorer outcomes observed with second-line treatment may reflect differences in prior treatment exposure and disease course compared with patients receiving CDK4/6 inhibitors in the first-line setting. Although fulvestrant use was associated with shorter PFS in univariable analysis, this finding should be interpreted cautiously because endocrine partner selection may partly reflect treatment history and clinical decision-making. Treatment history and prior endocrine exposure are recognized considerations in the selection of endocrine therapy in advanced HR+/HER2− breast cancer (31, 32). Additionally, the counterintuitive direction of the HR observed for ECOG PS 2 should be interpreted cautiously, given the small number of patients in this subgroup (*n* = 12) and the lack of statistical significance across the analyses. Several limitations should be acknowledged, including the retrospective design and limited availability of detailed nutritional and functional assessments, such as muscle strength, physical performance, and dietary intake. Formal intra-observer reproducibility of CT-based skeletal muscle measurements was not assessed. Additionally, most patients received ribociclib, while only a small proportion received palbociclib and none received abemaciclib; therefore, the findings may not be generalizable across all CDK4/6 inhibitors. Furthermore, because the study did not include a control group of patients not receiving CDK4/6 inhibitors, our findings support the prognostic value of sarcopenia in this treatment setting but do not establish sarcopenia as a predictive marker of benefit from CDK4/6 inhibition. Owing to the retrospective design, endocrine sensitivity or resistance could not be reliably characterized across the entire cohort. Longitudinal changes in skeletal muscle mass during treatment were not evaluated, and overall survival, dose intensity, treatment adherence, and detailed toxicity outcomes according to sarcopenia status were unavailable. The potential influence of unmeasured clinical, nutritional, and disease-related factors should also be considered. Nevertheless, the study included a clinically well-defined patient cohort with HR+/HER2− metastatic breast cancer treated with CDK4/6 inhibitors. Importantly, CT-defined sarcopenia was assessed using images routinely obtained for staging and treatment monitoring, without requiring additional imaging or radiation exposure. Its association with clinical outcomes therefore supports CT-based skeletal muscle assessment as a practical and readily accessible approach to risk stratification in routine oncology practice.

## 5 Conclusions

CT-defined sarcopenia was independently associated with shorter PFS in patients with HR+/HER2− metastatic breast cancer treated with CDK4/6 inhibitors. Because skeletal muscle assessment can be performed using CT images routinely obtained for staging and treatment monitoring, it may provide a practical and readily available approach to identifying patients at higher risk. Prospective studies are needed to validate these findings and determine whether interventions targeting sarcopenia improve clinical outcomes.

## Data Availability

The original contributions presented in the study are included in the article/Supplementary Material, further inquiries can be directed to the corresponding author.
